# Whole genome sequencing of *CCR5* CRISPR-Cas9-edited Mauritian cynomolgus macaque blastomeres reveals large-scale deletions and off-target edits

**DOI:** 10.3389/fgeed.2022.1031275

**Published:** 2023-01-12

**Authors:** Jenna Kropp Schmidt, Yun Hee Kim, Nick Strelchenko, Sarah R. Gierczic, Derek Pavelec, Thaddeus G. Golos, Igor I. Slukvin

**Affiliations:** ^1^ Wisconsin National Primate Research Center, University of Wisconsin-Madison, Madison, WI, United States; ^2^ University of Wisconsin Biotechnology Center, University of Wisconsin-Madison, Madison, WI, United States; ^3^ Department of Comparative Biosciences, University of Wisconsin-Madison, Madison, WI, United States; ^4^ Department of Obstetrics and Gynecology, University of Wisconsin-Madison, Madison, WI, United States; ^5^ Department of Pathology and Laboratory Medicine, University of Wisconsin-Madison, Madison, WI, United States; ^6^ Department of Cell and Regenerative Biology, University of Wisconsin-Madison, Madison, WI, United States

**Keywords:** CRISPR- Cas9, whole genome sequencing (WGS), embryo, macaque, CCR5

## Abstract

**Introduction:** Genome editing by CRISPR-Cas9 approaches offers promise for introducing or correcting disease-associated mutations for research and clinical applications. Nonhuman primates are physiologically closer to humans than other laboratory animal models, providing ideal candidates for introducing human disease-associated mutations to develop models of human disease. The incidence of large chromosomal anomalies in CRISPR-Cas9-edited human embryos and cells warrants comprehensive genotypic investigation of editing outcomes in primate embryos. Our objective was to evaluate on- and off-target editing outcomes in *CCR5* CRISPR-Cas9-targeted Mauritian cynomolgus macaque embryos.

**Methods:** DNA isolated from individual blastomeres of two embryos, along with paternal and maternal DNA, was subjected to whole genome sequencing (WGS) analysis.

**Results:** Large deletions were identified in macaque blastomeres at the on-target site that were not previously detected using PCR-based methods. *De novo* mutations were also identified at predicted CRISPR-Cas9 off-target sites.

**Discussion:** This is the first report of WGS analysis of CRISPR-Cas9-targeted nonhuman primate embryonic cells, in which a high editing efficiency was coupled with the incidence of editing errors in cells from two embryos. These data demonstrate that comprehensive sequencing-based methods are warranted for evaluating editing outcomes in primate embryos, as well as any resultant offspring to ensure that the observed phenotype is due to the targeted edit and not due to unidentified off-target mutations.

## 1 Introduction

Advances in genome editing, particularly using CRISPR-Cas9 technology, have facilitated the introduction and correction of disease-associated mutations in animal and cell culture models. Non-human primates (NHPs) are superior for modeling human diseases as they share similar aspects of immune, neuro-, and reproductive physiology and are ideal for transplant and neurodevelopmental disorder research. The interest in creating NHP models of human disease has been augmented by the need to better define the etiology of a disease and for the development of treatments and therapeutics (Dray et al., 2018; Abbott et al., 2019; Moshiri et al., 2019; Tapmeier et al., 2021; Ozirmak et al., 2022). For example, resistance to human immunodeficiency virus (HIV) has been observed in human patients with a 32 base pair deletion in the *CCR5* gene (*CCR5*-Δ32). CCR5 serves as an HIV co-receptor (Dean et al., 1996; Liu et al., 1996; Samson et al., 1996). Transplantation of hematopoietic stem cells (HSCs) containing the *CCR5*-Δ32 mutation to human HIV patients led to the cure of HIV infection in some but not all cases (Hütter et al., 2009; Allers et al., 2011; Henrich et al., 2014; Cummins et al., 2017). Generating NHPs with *CCR5*-deletions would aid in determining the mechanisms of HIV elimination following transplantation of allogeneic HSCs with *CCR5* mutations and the development of clinical protocols for reproducible HIV cure (Schmidt et al., 2022b).

Genome editing approaches to create gene disruption in NHP embryos have been successful, yet evidence of CRISPR-Cas9-induced chromosomal anomalies in mammalian cells and embryos warrants further investigation of embryonic editing outcomes in primate embryos. CRISPR-Cas9 editing has been shown to result in large scale deletions (up to 6 kb) and whole chromosome loss leading to genomic instability in mouse embryonic stem cells (Kosicki et al., 2018) and embryos (Adikusuma et al., 2018; Papathanasiou et al., 2021). CRISPR-Cas9 editing in human embryos has resulted in the loss of the targeted allele (Zuccaro et al., 2020) and also segmental chromosome losses (Alanis-Lobato et al., 2021). Moreover, large scale deletions in human embryos at an off-target site were also observed (Zuccaro et al., 2020). Loss of heterozygosity surrounding the on-target site is another consequence of CRISPR-Cas9 targeting observed in human embryos (Zuccaro et al., 2020; Alanis-Lobato et al., 2021). Collectively, these studies have revealed undesired on- and off-target mutations that arise when using wild-type Cas9 for gene correction in human and mouse embryos.

We previously demonstrated CRISPR-Cas9 editing of *CCR5* in Mauritian cynomolgus macaque (MCM, *Macaca fascicularis*) embryos using PCR-based methods to confirm successful targeting of the locus (Schmidt et al., 2020). The objective of the present study was to comprehensively evaluate on- and off-target editing in CRISPR-Cas9-edited MCM embryos using whole genome sequencing (WGS) methods to survey individual blastomeres. Molecular analysis revealed large-scale deletions contributing to greater mosaicism within individual embryos than was previously identified using PCR-based methods. Given that large-scale on- and off-target mutations might hinder establishment of a viable pregnancy, further optimization of macaque embryo editing to avoid targeting errors would be essential to facilitate generation of novel NHP models for human diseases.

## 2 Materials and methods

### 2.1 Animals

Methods for deriving the MCM embryos analyzed in the present study were previously reported (Schmidt et al., 2020). Parental DNA was obtained from a female (12 years) and male (6 years) MCM used in that study. All procedures were performed in accordance with the NIH Guide for the Care and Use of Laboratory Animals and under the approval of the University of Wisconsin-Madison College of Letters and Sciences and Vice Chancellor’s Office for Research and Graduate Education Institutional Animal Care and Use Committee.

### 2.2 Isolation of CRISPR-Cas9 injected embryo DNA

*In vitro* fertilized Mauritian cynomolgus macaque embryos were produced as previously described (Schmidt et al., 2020). Briefly, one-cell stage embryos were microinjected with Cas9 complexed with two sgRNAs targeting exon 2 of the *CCR5* gene to form the ribonucleoprotein (RNP). Embryos were cultured individually in a microwell of a CultureCoin MIRI-TL dish (Esco Medical, Denmark) containing 25 μL of Global medium overlaid with mineral oil and the culture dish was placed in a MIRI TL Time-Lapse incubator (Esco Medical) to monitor embryo development. Individual blastomeres from two embryos arrested at the 6-cell and 9-cell stage were isolated. The zona pellucida was removed by treatment with 1 mg/ml of activated pronase E (Sigma-Aldrich, cat no: P2730) and the embryo was washed in calcium- and magnesium-free PBS with .2% EDTA and 1 mg/ml human albumin (MP Biomedicals, cat no: 823051). Individual blastomeres were then dissociated by gently pipetting the embryo. DNA was amplified from individual blastomeres using a REPLI-G single cell kit (Qiagen, cat no: 150343).

### 2.3 PCR analysis of CCR5 on-target editing in individual blastomeres

PCR amplifications were performed as previously described to assess the targeted *CCR5* region (Schmidt et al., 2020) using primers that either amplified short (613 bp) or long (2,925 bp) amplicons surrounding the predicted cut sites. PCR products were run on 1.2%–1.5% agarose gels at 120 V. On-target *CCR5* editing was determined by visualizing either the expected wild-type (unmodified) *CCR5* PCR amplicon size of 613 bp or a biallelic mutation producing a product of 415 bp. To evaluate large-scale deletions near the on-target site, a long-range *CCR5* PCR was performed. PCR primer sequences and expected amplicon sizes are listed in Supplementary Table S1. PCR reactions were performed using the Q5 Hot start High-Fidelity DNA polymerase kit following manufacturer recommendations. Gel electrophoresis was performed using standard methods to visualize the amplicons.

### 2.4 Isolation of parental DNA

Blood draws from both the oocyte and semen donors of the in vitro-produced embryos were performed to obtain parental DNA from peripheral blood mononuclear cells. Genomic DNA was isolated from blood cells using a Quick-DNA Miniprep kit (Zymo Research, cat no: D3024).

### 2.5 DNA quality assessment

DNA quality was assessed at the University of Wisconsin Biotechnology Center’s by the NexGen DNA Sequencing Core using an Agilent Femto Pulse system (Agilent, Santa Clara, CA) to confirm a uniform yield of DNA product with the average product length of greater than 9.4 kb.

### 2.6 Whole genome sequencing and analysis

Whole genome sequencing was performed by the University of Wisconsin-Madison Biotechnology Center using the Illumina short-read platform and a NovaSeq 6000 instrument. Reads were trimmed to remove sequencing adapters and low quality base calls using the software skewer (Jiang et al., 2014) and then mapped to the *Macaca fascicularis* reference genome, M_fascicularis_5.0, using an Illumina Dynamic Read Analysis for GENomics (DRAGEN) Bio-IT platform version 3.7. Small variant and calling was performed using DRAGEN. Variants from control (parental) samples were used to filter and identify *de novo* mutations. Variant annotation was performed using SNEPeff tool that will predict synonymous or non-synonymous amino acid changes, gains or losses of start/stop codons, and frame shifts due to insertions or deletions. Structural analysis was performed using Parliament2 (Zarate et al., 2020) and only those called by at least two callers were included. Variants with lower quality that were filtered out as well as non-filtered variants are included as potential candidate mutations. *De novo* structural variants were those identified in blastomeres that were not present in the parental sequences. Short read sequencing is not ideally suited for calling structural variants, hence low quality-filtered out variants as well non-filtered variants are both included as potential candidates. Integrated Genomics Viewer software (https://software.broadinstitute.org/software/igv/download) was used to view the WGS data.

### 2.7 Off-target analysis

Potential CRISPR-Cas9 off-target regions were identified using the Cas-OFFinder tool (Bae et al., 2014) (http://www.rgenome.net/cas-offinder/) and allowing for three mismatches. Regions of interest were then evaluated in the WGS dataset to see if *de novo* mutations were present in individual blastomeres compared to the parental DNA. The presence of *de novo* mutations in three predicted off-target genes were assessed by Sanger sequencing of PCR amplicons containing the region of interest for individual blastomeres. DNA obtained from a wild-type cynomolgus macaque iPSC line was sequenced in parallel. PCR reactions were performed using the Q5 Hot start High-Fidelity DNA polymerase kit following manufacturer recommendations and the reactions were cleaned up using a Gel extraction and PCR clean up kit (IBI, cat no: IB47010). PCR primer sequences are listed in Supplementary Table S1. Sanger sequencing reactions were carried out by Functional Biosciences Inc., Madison, Wisconsin and the sequencing data was analyzed using the 4Peaks (https://nucleobytes.com/4peaks/index.html) application.

## 3 Results

### 3.1 WGS of individual blastomeres produces variable sequence coverage

**FIGURE 1:**
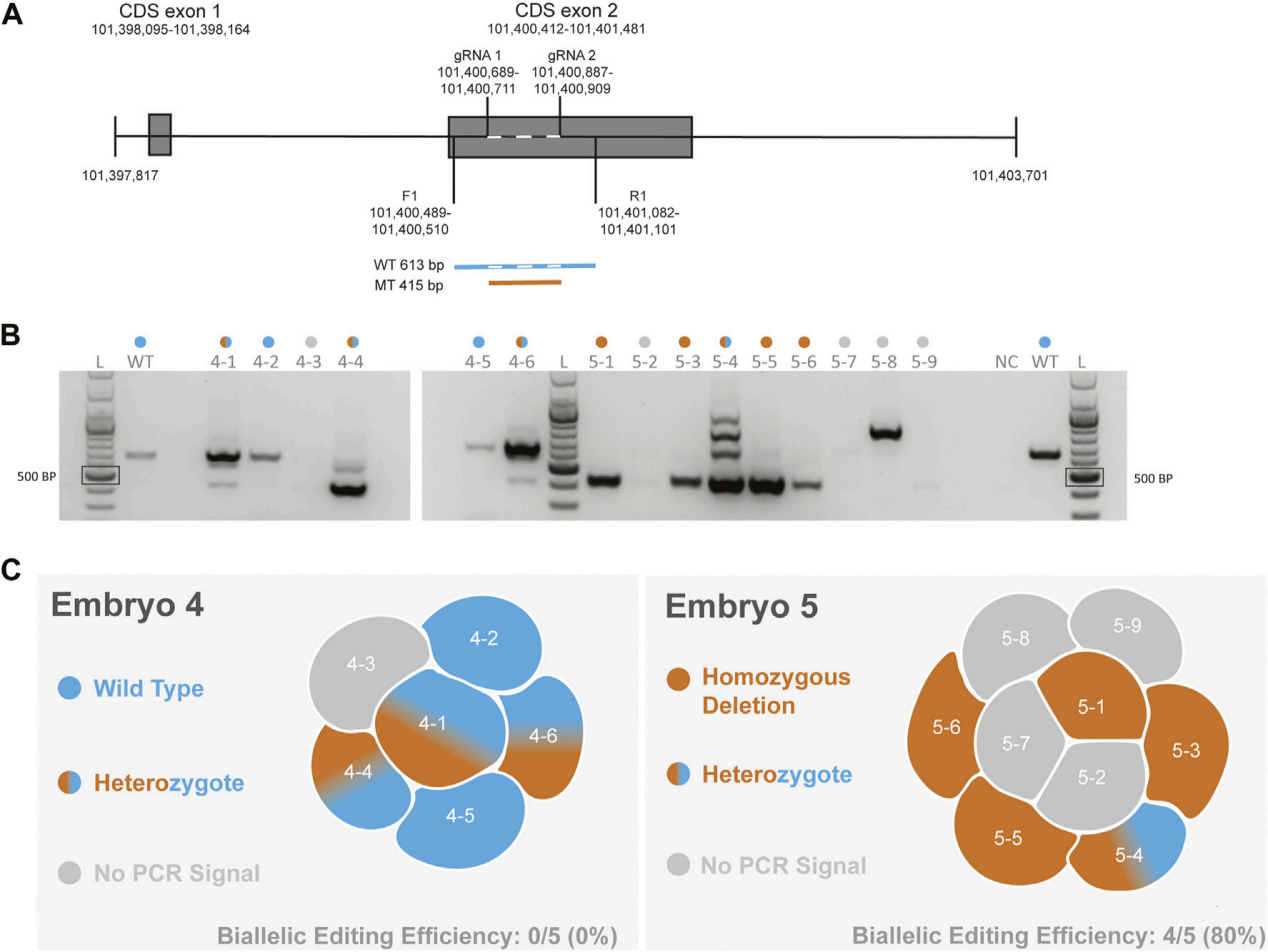
Evaluation of *CCR5* editing in MCM embryos using PCR-based methods **(A)**. Schematic of the *CCR5* gene including gRNA targeting and the forward (F1) and reverse (R1) primer. Wild-type (WT) product is 613 bp in length, whereas a biallelic deletion mutation (MT) produces a 415 bp product. Dashed line with the wild-type sequence indicates the targeting region **(B)**. Gel electrophoresis images of PCR products from blastomeres of embryos 4 and 5. A positive control reaction with DNA from an unmanipulated control embryo and a no template negative control (NC) were included. The colored dots above each lane indicate the editing outcome as indicated in 1C. The PCR and gel electrophoresis results were provided in our initial report describing *CCR5* editing in MCM embryos (Schmidt et al., 2020) **(C)**. Diagram summarizing the PCR-based editing outcome and biallelic editing efficiency within each embryo.

To functionally delete *CCR5* in macaque embryos, we designed gRNAs that would encompass a 24-bp deletion that has previously been shown to be essential for expressing *CCR5* in non-human primates (Chen et al., 1998). Our previous cell-based editing experiments confirmed successful on-target editing with functional deletion of the *CCR5* gene in both human (Kang et al., 2015) and macaque (D'Souza et al., 2022) iPSCs. A schematic diagram of the targeting region is shown in Figure 1A. In our initial report describing targeting of this region in MCM embryos, PCR-based methods were used to evaluate CRISPR-Cas9 targeting of the *CCR5* locus (Schmidt et al., 2020). Two embryos were dissociated into individual blastomeres and DNA was isolated for PCR evaluation and single-cell WGS. PCR and gel electrophoresis revealed editing mosaicism in each embryo (Figures 1B,C), although PCR signal was undetected in one and three blastomeres from embryo 4 and 5, respectively (Figure 1B).

**FIGURE 2:**
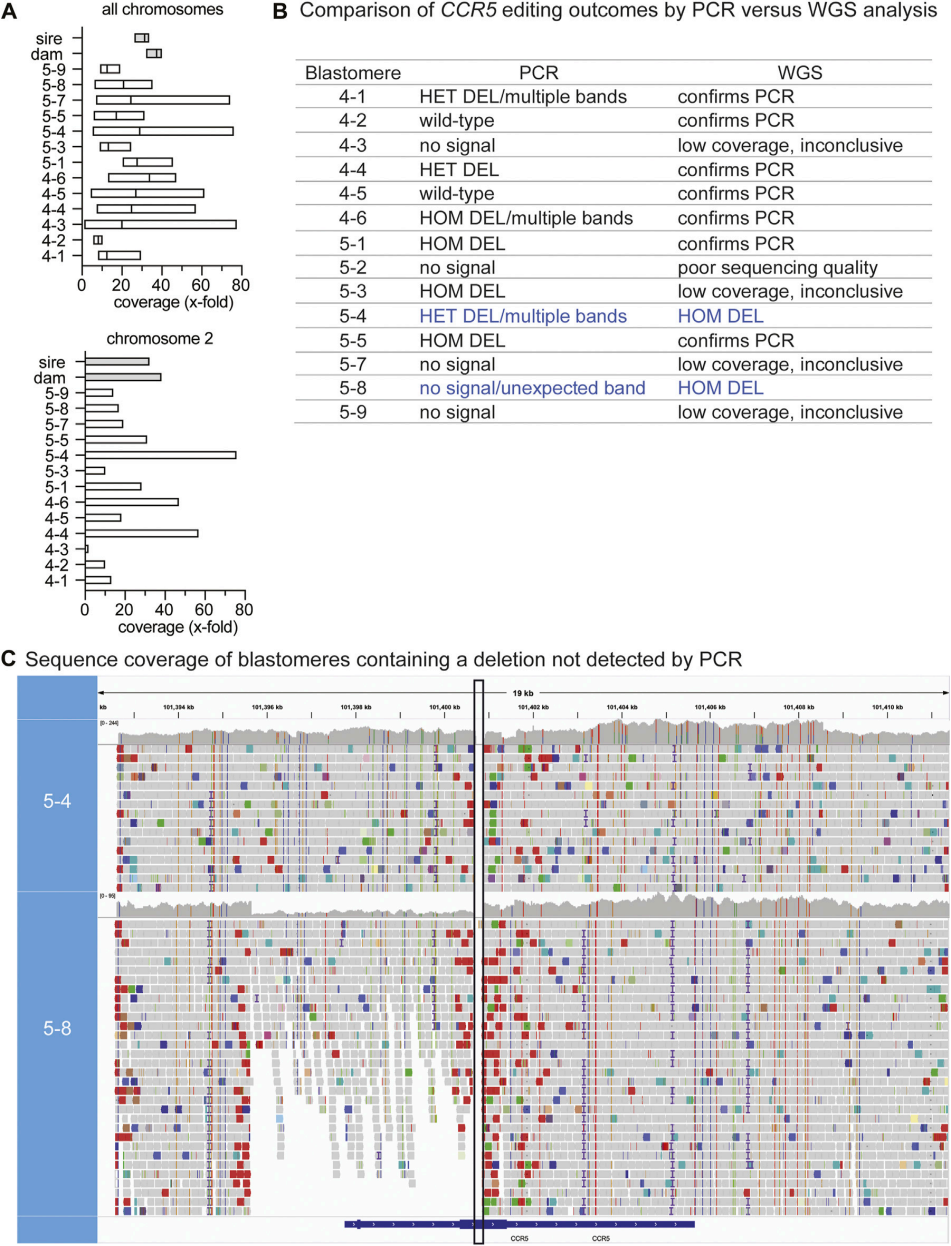
WGS identification of genotypes not identified by PCR-based methods **(A)**. The mean and range in sequencing coverage depth (x-fold coverage) are plotted for each blastomere and parental DNA. The mean and range for all chromosomes is shown in the upper graph and the lower graph shows chromosome 2; *CCR5* resides on chromosome 2 **(B)**. Comparison of *CCR5* editing outcomes by PCR versus WGS analysis. Blue text indicates cells where WGS identified deletions not previously identified by PCR. HET: heterozygous, HOM: homozygous **(C)**. Sequence coverage at the *CCR5* targeting region in blastomeres 5-4 and 5-8 where homozygous/biallelic edits were observed by WGS. The WGS viewer software indicates potential deletions with red bars and in the alignment tracks of 5-8, these are present around the target region and were minimal to absent in the parental coverage map. The vertical black box indicates the expected deleted region between the gRNA target sites.

Single-cell DNA amplification and WGS was performed on DNA from all six blastomeres of embryo 4 and 8 of 9 blastomeres from embryo 5. In addition, DNA isolated from peripheral blood mononuclear cells from the sire and dam of the embryos was sequenced in parallel. Chromosomal coverage varied across individual blastomeres and chromosomes ranging from .81–77.77-fold coverage, whereas the parental sequence coverage was at a depth of ∼30x, as expected for somatic cells. A sequencing coverage of 30x is interpreted as the genome was sequenced ∼30 times. Figure 2A shows the mean coverage and range of sequence depth across chromosomes for each sample and illustrates the variability in coverage in blastomeres compared to parental DNA isolated from peripheral blood cells. The *CCR5* gene resides on chromosome 2, a chromosome that greatly varied in sequence depth coverage across blastomeres (Figure 2A). Sequence coverages for each sample by chromosome are listed in Supplementary Table S2. Blastomere 5-2 had an atypical distribution of GC content and was excluded from all analyses. For the remaining blastomeres, the sequence coverage at on- and off-target regions was taken into consideration and when limited sequences were observed at the region of interest the WGS result was deemed as not conclusive.

### 3.2 WGS identified additional on-target deletions

WGS confirmed the genotypes identified using PCR-based methods for most of the blastomeres that had detectable PCR signal (7 out of 9) and determined the genotype for one blastomere in which the *CCR5* region could not be amplified by PCR (Figure 2B). Sequences that spanned the target region are indicative of wild-type sequences, whereas a deletion was inferred if there was a break in the sequence coverage. Representative examples of wild-type (WT), heterozygous (HET) and homozygous deletion (HOM DEL) genotypes as determined by WGS are illustrated in Supplementary Figure S1. WGS sequence coverage at the *CCR5* targeting region also identified deletions not previously observed using PCR-based methods (Figure 2B). If there was poor sequence coverage at the target site, the WGS genotype could not be determined and was deemed inconclusive. Blastomere 5-4 was identified to be HET by PCR-based methods, however, when looking at the sequencing alignment, no sequences spanned the region between the gRNA sites indicating that the blastomere contained a biallelic deletion (Figure 2C). Regardless of the unexpected gel band pattern of 5-4, the HOM DEL was confirmed *via* Sanger sequencing of the amplicons isolated from the three lowest bands in the agarose gel; each amplicon contained the expected ∼200 bp deletion and no WT sequences were detected (Supplementary Material S2). PCR using the standard primer pair previously failed to identify the genotype of blastomere 5-8, whereas WGS coverage indicated deletions spanning the gRNA sites and revealed large-scale deletions that encompassed the PCR primer sequences (Figure 2C). Moreover, the depth of coverage was reduced by approximately half at the 5’ end, confirming that one allele contained a large-scale deletion upstream of the first gRNA site.

### 3.3 Identification and validation of large-scale deletions at the CCR5 targeting region

*De novo* structural variants that were not present in the parental DNA but were within individual blastomeres of both embryos 4 and 5 were identified by WGS (Table 1). As short read sequencing platforms are not ideally suited for identifying structural variants, those that were called in at least two variant callers are listed. The on-target deletion between the gRNA sites was identified as a variant in 4-4. Large-scale deletions that span the target region were identified in blastomeres 4-6, 5-4, and a similar deletion of ∼5.2 kb was seen in both 5-5 and 5-8. Several inversions were also identified and within each embryo an inversion was unique to a pair of blastomeres.

**TABLE 1:** *De novo* structural variants at the *CCR5* locus identified in individual blastomeres.

	Blastomere	Variant type	Position	Size (kb)
Embryo 4	4-4	inversion	25,740,108–123,812,023	98,071.915
		inversion	91,767,007–127,108,482	35,341.475
		deletion	101,400,706–101,400,904	.198
	4-5	inversion	25,740,424–123,812,038	98,071.915
	4-6	duplication	82,604,203–135,851,822	53,247.619
		deletion	101,400,160–101,400,916	.756
Embryo 5	5-3	inversion	51,210,725–141,809,530	90,598.805
	5-4	deletion	14,388,873–142,671,745	128,282.872
		inversion	91,766,840–127,108,372	35,341.532
	5-5	inversion	91,766,814–127,108,375	35,341.561
		deletion	101,395,673–101,400,914	5.214
	5-8	deletion	101,395,673–101,400,914	5.214
		deletion	101,400,695–101,401,620	.925

**FIGURE 3:**
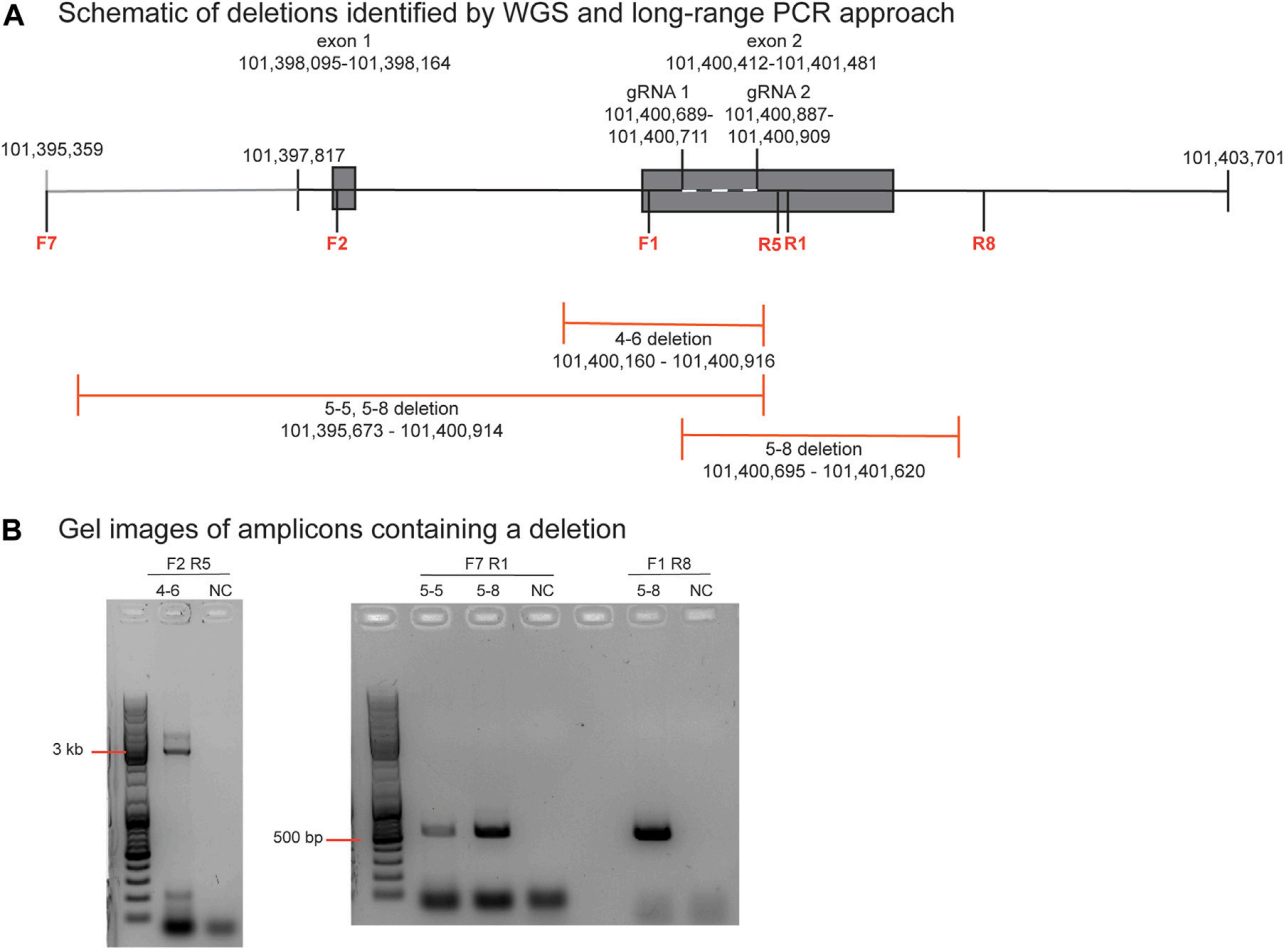
Identification and verification of a large-scale deletion identified by WGS in blastomere 4-6 **(A)**. Schematic diagram of deletions identified by WGS in blastomeres 4-6, 5-5, and 5-8, and the positions of the primers to evaluate the ∼756 bp deletion in blastomere 4-6. Primers are denoted as F or R and their sequences are listed in Supplementary Table S1 **(B)**. Gel electrophoresis image of the PCR amplicons generated by primers that flank each deletion. A no template negative control (NC) reaction was also included.

The PCR primer sequences used for initially genotyping blastomeres were within the deleted regions, hence these deletions could not be identified using PCR-based methods. The positions of the large-scale deletions identified by WGS in blastomeres 4-6, 5-5, and 5-8 are illustrated in Figure 3A. PCR-based methods using primers flanking each deleted region followed by gel electrophoresis confirmed the presence of the large-scale deletions in 4-6, 5-5, and 5-8 (Figure 3B). The large-scale deletions were confirmed by Sanger sequencing of PCR amplicons for the deletions detected in 4-6, 5-5, and 5-8 with one exception (Supplementary Material S1). Poor sequencing data did not allow for verification of the ∼5.2 kb deletion in the 5.8 blastomere whereas the deleted sequence was confirmed in blastomere 5.5 (Supplementary Material S1).

### 3.4 WGS detection of on-target INDELs in blastomere 5-4

**FIGURE 4:**
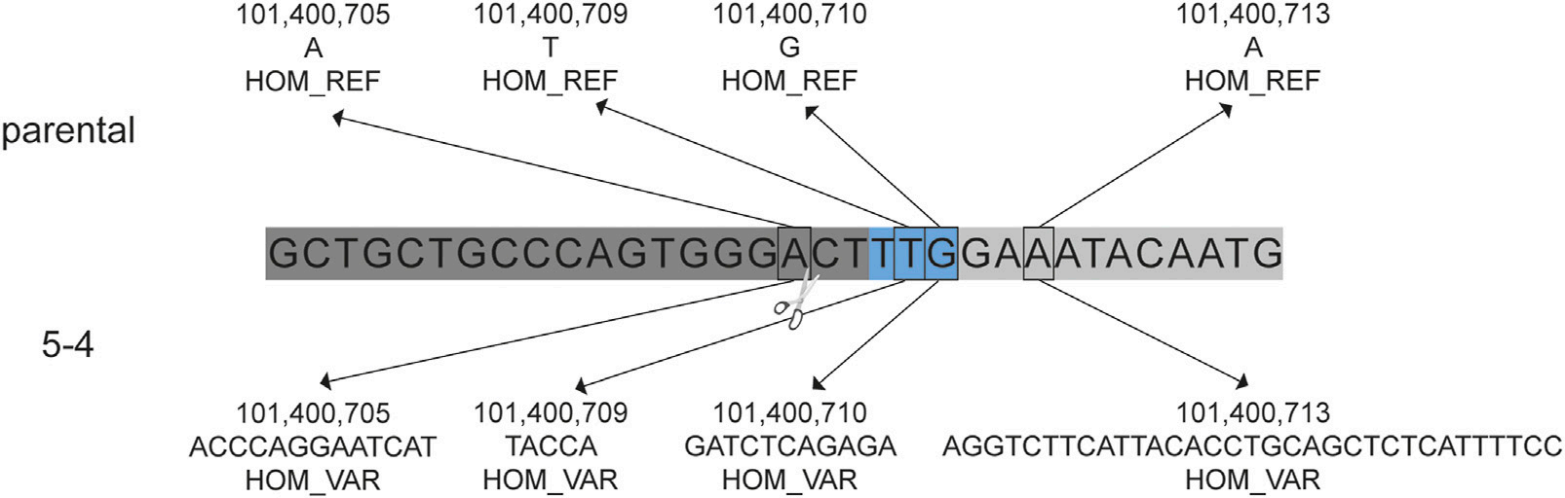
Insertions in blastomere 5-4 at the CCR5 target site. *De novo* insertions identified in blastomere 5-4 that were not identified in the parental DNA. The gRNA 1 sequence is indicated by the dark gray bar and the blue indicates the PAM sequence (5′-TTG). Homozygous (HOM) variants (VAR), specifically insertions, were detected near the predicted DNA cut site (indicated by scissors) that were not detected in the cynomolgus macaque reference genome (REF) nor the parental DNA sequence.

The presence of insertions and/or deletions (INDELs) within the on-target region was evaluated. Single nucleotide variants (SNVs) were identified and considered to be *de novo* mutations if they were present in the blastomere but not detected in either parent. The number of *de novo* SNVs detected in each blastomere is provided in Supplementary Table S3. Homozygous insertions of 13 bp, 5 bp, 11 bp, and 33 bp were identified near the gRNA one cut site in blastomere 5-4 that were not identified in the cynomolgus macaque reference genome nor the parental DNA sequences (Figure 4). This was the only blastomere with INDEL formation near a cut site.

### 3.5 Off-target edits detected at loci with restricted sequence homology to the gRNAs

To assess the feasibility of detecting off-target mutations by WGS, we utilized the *in silico* Cas-OFFinder tool to identify off-target sites based on sequence homology to the gRNA sequences and the total number of off-target sites when allowing for up to 9 mismatches between the gRNA and off-target sequence is provided in Supplementary Table S4. While off-target editing can occur at sites with up to 4 mismatches (Pattanayak et al., 2013; Cromer et al., 2022), we focused on assessing the WGS data at 93 predicted off-target regions that contained 2 or 3 mismatches which occurs more commonly. *De novo* mutations within individual blastomeres were identified by WGS in 16 predicted off-targeted sites of which 7 were located within genes and nine were located in intergenic regions (Table 2). The specific mutations identified by WGS at each off-target site are provided in Supplementary Material S2.

**TABLE 2:** *De novo* mutations identified by WGS in predicted off-target regions.

	Chr	Position	Gene	Sequence (5′-3′)	Blastomeres with *de novo* variants
gRNA 1	2	101,400,689–101,400,711	CCR5*	GCTGCT​GCC​CAG​TGG​GAC​TT	
	1	63,497,146–63,497,168	*NFASC*	T C A ​GCT​GCC​CAG​TG T ​GAC​TT	4-4, 4-5, 5-3, 5-4, 5-7, 5-8, 5-9
	1-scaffold	378,063–378,085	intergenic_ke145507.1	GCT​GCT​GCC​CAG​T A G​ TG C​TT	4-4, 4-6
	10	31,267,650–31,267,672	intergenic_CM001928.1	T CT​GCT​GCC​CAG​TGG​G C C​T C	5-1
	19	40,019,282–40,019,304	*DLL3*	GC CC CT​GC A ​CAG​TGG​GAC​TT	4-4, 4-6, 5-3
gRNA 2	2	101,400,887–101,400,909	CCR5*	GCT​GTG​TTT​GCC​TCT​CTC​CC	
	7	35,664,631–35,664,653	*LIPC*	GCT​GTG​ C TT​GCC​TCT​ T TC​CC	4-5, 4-6, 5-1
	9	7,497,178–7,497,200	*SFMBT2*	GCT​G GC ​TTT​GCC​TCT​CTC​CC	4-3, 4-6, 5-5, 5-7, 5-8
	11	129,547,583–129,547,605	intergenic_CM001929.1	GCT​GTG​ C TT​ C CC​TCT​CTC​CC	4-5, 4-6, 5-1, 5-5
	14	14,725,950–14,725,972	intergenic_CM001932.1	GCT​GTG​TT CT CC​TCT​CTC​CC	4-5, 4-6, 5-4
	1	199,634,457–199,634,479	intergenic_CM001919.1	GCT​GTG​ G TT​ T CC​TCT​CTC​C T	5-3
	3	53,360,905–53,360,927	intergenic_CM001921.1	C CT​GTG​ A TT​ A CC​TCT​CTC​CC	4-3, 4-5, 5-1, 5-5
	4	2,457,033–2,457,055	intergenic_CM001922.1	GCT​GTG​T C T​ C CC​TCT​C C C​CC	5-1
	5	188,649,889–188,649,911	intergenic_CM001923.1	AA T​GTG​TTT​GCC​TCT​ T TC​CC	4-3, 5-1, 5-4, 5-7, 5-8, 5-9
	7	28,263,588–28,263,610	*CYP19A1*	G GA ​GTG​TT G ​GCC​TCT​CTC​CC	5-7
	12	62,901,723–62,901,745	intergenic_CM001930.1	GCT​GTG​T CC ​GCC​TCT​CTC​ G C	4-1, 4-3, 5-3, 5-5, 5-8
	17	62,039,482–62,039,504	*NDFIP2*	GCT​GT C ​TTT​GCC​ A CT​CTC​ A C	4-3
	19	49,110,480–49,110,502	*CCDC114*	GCT​GTG​ CA T​ C CC​TCT​CTC​CC	5-1, 5-3, 5-4, 5-8

A total of 93 regions were assessed that contained 2-3 mismatches. *Denotes on-target gene sequence. Chr: chromosome.

Blastomeres with mutations at off-target regions identified by WGS were subjected to PCR and Sanger sequencing, with the exception that all blastomeres of embryo 5 were sequenced for the *SFMTB2* region. Sanger sequences are shown in Table 3. The T to G point mutation at the predicted region in the *NFASC* gene was identified in blastomeres of both embryos 4 and 5. This mutation is likely a spontaneously occurring mutation as the parental genotype was T/T, embryo 1 blastomeres were G/G and embryo 5 blastomeres carried either a T/T, G/G or T/G genotype. *De novo* deletions near the predicted cut site of the off-target regions in the *SFMBT2* and *LIPC* genes suggests off-target editing by the CRISPR-Cas9 RNP (Table 3). Furthermore, blastomeres of both embryos show deletions in *SFMBT2* and pairs of blastomeres in embryo 5 have the same 7 or 8 bp deletion suggestive that the editing occurred in a previous cell division similar to mutations introduced by on-target CRISPR-Cas9 targeting.

**TABLE 3:** Sanger-sequencing of amplicons containing predicted off-target mutations identified by WGS.

Gene	Sample	Sequence (5′-3′)
*NFASC*	WT	TCA​GCT​GCC​CAG​TGT​GACTT**GGG**
	4-4	TCA​GCT​GCC​CAG​ G GT​GAC​TT**GGG**
	4-5	TCA​GCT​GCC​CAG​ G GT​GAC​TT**GGG**
	5-3	TCA​GCT​GCC​CAG​TGT​GAC​TT**GGG**
	5-4	TCA​GCT​GCC​CAG​ G GT​GAC​TT**GGG**
	5-7	TCA​GCT​GCC​CAG​ G GT​GAC​TT**GGG**
	5-8	TCA​GCT​GCC​CAG​ G GT​GAC​TT**GGG**
	5-9	TCA​GCT​GCC​CAG​ G GT​GAC​TT**GGG**
*SFMBT2*	WT	GCT​GTG​TTT​GCC​TCT​CTCCC**AGG**
	4-3	GCT​GGC​TTT​GCC​TCT​C–––– G **GG**
	4-6	G N TGG N TTTGCCTCTCT N CC**AGG**
	5-1	NNN G NN TT NN C NNNNNCN CC**AGG**
	5-3	GCT​G GC ​TTT​G––––––––CC**AGG**
	5-4	NNG G CN TT NN C A T T T T TCCC**AGG**
	5-5	GCT​G GC ​TTT​G––––––––CC**AGG**
	5-6	NNN G GC TTT N CCT T T T TCCC**AGG**
	5-7	GCT​G GC ​TTT​GCC–––––––C G **GG**
	5-8	G NN G GA TTTGCC–––––––C G **GG**
*LIPC*	WT	GCT​GTG​CTT​GCC​TCT​TTCCC**TGG**
	4-5	GCTGTG ––– G ––––– N T NN C**TGG**
	4-6	G NN G N GC NN GC N TCTTTCCC**TGG**
	5-1	GCT​GTG​CTT​GCC​TCT​TTC​CC**TGG**

WT, wild-type sequence; underline text: predicted cut site; bolded font: PAM, sequence; red font: denotes mutation; dash(-): deletion; N, not conclusive base determination.

### 3.6 Structural variants detected at off-target sites

The presence of structural variants at predicted off-target sites was investigated for the off-target regions in the *NFASC*, *SFMBT2,* and *LIPC* genes. Large-scale deletions, inversions and duplications were identified at these sites by WGS (Table 4). An inversion and deletion at the *NFASC* off-target region was observed in each blastomere of embryos 4 and 5, and an inversion identified at the *LIPC* site was shared between blastomeres 4-5 and 4-6. A smaller deletion of 210 bp was detected in blastomere 4-1. Fewer off-target structural variants were shared among blastomeres within an embryo compared to those that were identified at the on-target site. A summary of on- and off-target mutations identified by WGS in each blastomere is provided in Supplementary Table S5.

**TABLE 4:** *De novo* structural variants at predicted off-target sites identified in individual blastomeres by WGS.

Gene	Blastomere	Variant type	Position	Size (kb)
*NFASC*	4-3	deletion	60,461,014–144,877,317	84,416.3
	4-4	inversion	29,288,909–135,582,062	103,293.2
		inversion	40,415,565–127,456,502	87,040.9
	5-3	inversion	44,728,954–174,674,237	129,945.3
	5-4	inversion	40,415,565–127,456,502	87,040.9
	5-7	deletion	63,036,915–113,225,838	50,188.9
	5-8	duplication	32,797,257–199,946,823	167,149.6
		deletion	60,460,921–144,877,320	84,416.4
*SFMBT2*	4-3	duplication	4,656,592–131,420,663	126,764.1
		duplication	631,150–44,472,044	43,840.9
		deletion	6,341,129–44,439,744	38,098.6
*LIPC*	4-1	deletion	35,664,439–35,664,648	0.2
	4-3	inversion	18,877,162–149,973,064	131,095.9
	4-4	inversion	22,447,357–70,039,192	47,591.8
	4-5	inversion	29,303,656–142,197,757	112,894.1
	4-6	duplication	1,727,262–94,864,685	93,137.4
		inversion	29,303,656–142,197,757	112,894.1
	5-1	deletion	27,706,507–56,330,973	28,624.5
	5-5	duplication	9,799,825–73,825,441	64,025.6
	5-7	inversion	18,272,609–49,503,847	31,231.2
	5-9	inversion	1,842,595–98,494,590	96,652.0

## 4 Discussion

In the present study, comprehensive assessment of CRISPR-Cas9 targeting in MCM blastomeres by WGS confirmed editing mosaicism, revealed undesired on- and off-target editing events in NHP embryos, including large scale deletions, and resolved genotypes at the on-target sites that were previously undetected using PCR-based methods. INDELs were observed at on- and predicted off-target sites, where sequence disruption was confirmed by Sanger sequencing for two off-target regions. WGS analysis also provided insight into the timing of CRISPR-Cas9 targeting as identical structural variants and *de novo* mutations were shared in pairs of blastomeres but were not identified in the majority of blastomeres suggesting that editing was delayed and did not occur at the one-cell stage. While CRISPR-Cas9 can introduce mutations at disease-associated loci in NHP embryos, the occurrence of unexpected editing events requires rigorous assessment of not only embryos, but also the offspring to confirm that any resulting phenotype is not due to off-target effects.

Undesired editing events at the on-target site, including large-scale deletions, have been observed in human and mouse embryos targeted with wild-type Cas9 nuclease (Adikusuma et al., 2018; Zuccaro et al., 2020; Alanis-Lobato et al., 2021; Papathanasiou et al., 2021). In the present study, the expected 198 bp deletion as well as 756 bp, 925 bp and ∼5.2 kb deletions were detected at the *CCR5* on-target site through WGS structural variant analysis of individual NHP embryonic cells. Deletions have been previously described in reports of CRISPR-Cas9 targeting in NHP embryos that were transferred to surrogates and produced edited offspring, including an ∼11.5 kb deletion in *SHANK3* in one cynomolgus monkey (Zhao et al., 2017), a ∼7.2 kb deletion in *PINK1* in two rhesus monkeys (Yang et al., 2019), and 920 bp at the *OCT4* knock-in site in a cynomolgus monkey (Cui et al., 2018). The *SHANK3* mutant died *in utero* at 125 days of gestation (term is 165 days) and the two *PINK1* mutants were triplets that died days after birth. These studies demonstrated that implantation and pregnancies can be achieved despite the presence of large-scale editing errors. Furthermore, on-target deletions have now been observed across studies in both NHP embryos and tissues where different genes were targeted by wild-type CRISPR-Cas9, which necessitates refinement of genome editing tools for creating precise disease-associated mutations.

In the current study, we identified pairs of blastomeres of the same embryo which contained similar structural variants, however these variants were not identical suggesting that they arose from separate editing events (e.g., blastomeres 5-5 and 5-8 shared a ∼5.2 kb deletion and 5-4 and 5-5 shared a ∼35,341 kb inversion near the *CCR5* targeting site). Mosaicism in blastomere genotypes suggests that editing was delayed and did not occur at the one-cell stage as the deletion was not detected in all cells of the embryo. Editing mosaicism has been observed in tissues of edited NHPs produced from embryo transfer of CRISPR-Cas9 targeted embryos (Niu et al., 2014; Chen et al., 2015; Tsukiyama et al., 2019; Zhou et al., 2019).

A goal of the present study was to assess the feasibility of using WGS to assess off-target effects in individual blastomeres. An *in silico* based approach guided the nomination of potential off-target regions based on sequence homology to the gRNAs allowing up to three mismatches. Following *CCR5* targeting, mutations were observed in the genes *SFMBT2* and *LIPC* by WGS and the introduction of INDELS was confirmed by Sanger sequencing. Blastomeres of both embryos displayed sequence disruption *via* INDEL formation with the presence of 4, 7 or 9 bp deletions in some of the cells at the predicted off-target site within the *SFMBT2* gene. These results confirmed that off-target editing could be assessed by WGS in individual blastomeres, although we used a biased *in silico* method that relied on assessing targets with sequence homology and did not evaluate potential targets with greater than three mismatches. Additional *in silico* nominated targets should be evaluated to fully assess the impact of off-target editing as CRISPR-Cas9 cleavage can occur at off-target sites with up to four mismatches (Pattanayak et al., 2013; Cromer et al., 2022). Moreover, unbiased methods that survey the whole genome without prior knowledge or prediction of sequence homology would be more informative, yet there is not a current superior method or technique for this analysis (Chaudhari et al., 2020; Atkins et al., 2021). *In vitro*-based off-target analysis methods in the future could be adapted for single-cells, but with current use of whole genome amplification (WGA) and an incomplete reference genome, a large number of false positives might be called due to errors incurred during WGA or due to differences in the reference assembly.

Relatively few off-target mutations have been identified in studies that have generated edited NHP offspring by transfer of CRISPR-Cas9 targeted embryos to surrogate embryo recipients. A 2 bp deletion in one off-target region was reported in an edited cynomolgus monkey (Cui et al., 2018) and one intronic and two intergenic INDELS were identified in two edited rhesus monkeys (Wang et al., 2018). Luo et al. (2019) identified *de novo* mutations that the authors thought were not introduced by CRISPR-Cas9 and rather could be attributed to natural spontaneous generational mutations or that were due to technical noise. In these previous NHP studies, WGS analysis was performed on DNA obtained from cells or tissues of live offspring or miscarried fetuses whereas here we reported WGS on individual blastomeres of NHP embryos. The higher incidence of off-target editing in this study could be explained by the editing efficiency of the RNP or it is possible that such significant off-target errors could have been embryonic lethal and therefore not present in offspring that survived to or near term. Editing has shown to be more rapid and efficient when targeting with an RNP versus Cas9 mRNA in NHP embryos (Midic et al., 2017). To mitigate potential off-target editing events, the following strategies could be implemented: 1) microinjection of the RNP at the time of fertilization (Lamas-Toranzo et al., 2019), 2) use of a Cas9 nuclease modified to improve specificity (Huang et al., 2022), or 3) use of a base or prime editing approach that does not result in a double-stranded DNA break (Zeballos and Gaj, 2020).

Off-target INDELs and segmental chromosome errors introduced by CRISPR-Cas9 have been observed in human embryos (Zuccaro et al., 2020). The formation of INDELs has been reported at a predicted off-target site that had two mismatches to the gRNA sequence (Zuccaro et al., 2020). Moreover, segmental chromosome errors were detected near predicted off-target sites and were often restricted to one cell, hence the authors concluded that the events likely occurred during the second or third cell cycle. While the present study did not focus on whole or segmental chromosomal errors, structural variants were detected at off-target sites that could be investigated in the future.

Large-scale mutations introduced by CRISPR-Cas9 editing in human and mouse embryos have shown to contribute to genomic instability through segmental and whole chromosomal loss (Adikusuma et al., 2018; Zuccaro et al., 2020; Alanis-Lobato et al., 2021; Papathanasiou et al., 2021). Unrepaired double-stranded DNA breaks at the CRISPR-Cas9 cleavage site have shown to result in fragmented chromosomes leading to chromosome mis-segregation and micronuclei formation in human cell lines and mouse embryos (Leibowitz et al., 2021; Papathanasiou et al., 2021). In human cleavage stage embryos, failure to replicate the genome before entry into mitotic divisions contributes to poorer embryo quality due to aneuploidy associated with chromosomal fragmentation and the formation of a micronucleus (Palmerola et al., 2022). Human and NHP embryos naturally have a higher incidence of aneuploidy where partial or whole chromosomes encapsulated by micronuclei may be present in fragmented cells of the embryos (Daughtry et al., 2019; Palmerola et al., 2022). Cellular fragmentation was previously observed in >65% of *in vitro* fertilized cleavage stage rhesus macaque embryos, and when analyzing individual blastomeres from 50 embryos, 73.5% showed whole and/or partial chromosomal losses or gains (Daughtry et al., 2019). The incidence of chromothripsis was not assessed in the present study given the limitations in interpreting WGA artifacts in light of an incomplete reference genome, in addition to the difficulty in discerning whether chromosome loss was due to CRISPR-Cas9 targeting or naturally-occurring cellular fragmentation events. As CRISPR-Cas9 editing errors can result in chromosomal disruption and/or elimination (Leibowitz et al., 2021; Papathanasiou et al., 2021) it is plausible that the embryo development may be negatively impacted and could explain the poor embryo transfer rate observed in our previous study (Schmidt et al., 2020) and in general the low live birth rates of CRISPR-Cas9 targeted NHPs (Schmidt et al., 2022a; Schmidt et al., 2022b).

### 4.1 Limitations of the study

Single-cell WGA can introduce amplification bias potentially limiting the interpretation of the variants identified in this study. Several studies comparing commercially available sc-WGA kits revealed differences in reproducibility, error rates, target coverage, read depth distribution and allele drop out, however, the REPLI-G sc-WGA kit used in the current study was shown to have a high mapping rage (>90%), be reproducible and have a lower error rate (Borgström et al., 2017; Biezuner et al., 2021) compared to other kits. In the present study, sequence coverage was variable across chromosomes within and across individual blastomeres compared to the parental DNA that did not undergo WGA. A limitation to the present study is the lack of analysis of unmanipulated control blastomeres to assess the rate of errors or artifacts incurred due to WGA. Translocation events were not called for this reason and only structural variants identified by two callers were reported in this study. While it is uncertain whether the structural variants identified by WGS are due to CRISPR-Cas9 targeting, the variants were detected in pairs of blastomeres that were processed through independent WGA reactions suggesting that the mutation occurred during an early cleavage division or that similar sites of the genome are reproducibly prone to amplification errors during the WGA process. Recent advances in WGA technology such as primary template-directed amplification (Gonzalez-Pena et al., 2021) or linear amplification through transposon insertion (Chen et al., 2017) have shown to amplify single-cell genomes with more uniformity and accuracy. While these technologies are not incorporated into a commercial kit, they could be implemented in future single blastomere WGS studies for greater accuracy and reproducibility.

## 5 Conclusion

Overall, utilizing a WGS approach to determine CRISPR-Cas9 editing outcomes allows for the identification of edits not identified by PCR. In this study, WGS revealed the incidence of on-target large-scale deletions and INDEL formation at off-target sites. Imprecise editing could hinder the development of an NHP disease model that both genocopies and phenocopies the disease. The consequences of undesired editing events on gene expression of off-target and/or neighboring genes was not evaluated in this study, but should be considered in future studies. Based on evidence from human and mouse embryonic targeting by CRISPR-Cas9, it is likely that chromosomal damage incurred early in embryo development could negatively impact embryo viability. It remains unclear whether a reduced concentration or volume of the RNP delivered to the one-cell embryo would have a dose-dependent impact on on-target errors. Additional studies are needed to optimize embryonic editing by wild-type Cas9 and/or to use alternative next-generation Cas9 nucleases that do not create a double-stranded DNA break (Komor et al., 2016; Zeballos and Gaj, 2020). Regardless, WGS analysis should be implemented to thoroughly characterize editing genotypes in NHP models generated through this technology.

## Data Availability

The datasets presented in this study can be found in online repositories. The names of the repository/repositories and accession number(s) can be found below: https://www.ncbi.nlm.nih.gov/, PRJNA880597.

## Abbreviations

WGS, Whole genome sequencing; NHP, Non-human primate; HIV, Human immunodeficiency virus; HSC, Hematopoietic stem cell; MCM, Mauritian cynomolgus macaque; RNP, Ribonucleoprotein; CNV, copy number variation; WT, Wild-type; HET, Heterozygous; HOM, Homozygous; DEL, Deletion; InDel, Insertions and/or deletions; SNVs, Single nucleotide variants.
