# Supplementary material for: Whole genome sequencing of CCR5 CRISPR-Cas9-edited Mauritian cynomolgus macaque blastomeres reveals large-scale deletions and off-target edits
Source: Front Genome Ed. 2023 Jan 12;4:1031275. doi: 10.3389/fgeed.2022.1031275 (PMC9877282; doi:10.3389/fgeed.2022.1031275)
Supplement: Supplementary file 1 [file Table1.docx]

| Gene | Forward (5’-3’) | Reverse (5’-3’) | Amplicon Size (bp) | |
| --- | --- | --- | --- | --- |
| *CCR5* on-target | | | | |
| F1/R1 | TCAATGTGAAACAAATCGCAGC | TCGTTTCGACACCGAAGCAG | WT: 613 | DEL: 415 |
| F2/R5 | GCACAACTCATCTGCCAGAAG | AGCCCCAAGATGACCATC | WT: 2,925 | DEL: 2,165 |
| F1/R8 | TCAATGTGAAACAAATCGCAGC | CCCATATGTTGCCTTGTACAAG | WT: 1,409 | DEL: 484 |
| F7/R1 | AGACATGGAGCAGCTGCTTAA | TCGTTTCGACACCGAAGCAG | WT: 5,473 | DEL: 499 |
| Off-target | | | | |
| *LIPC* | CATTTACTGGCAAGCTGAGC | GTCAGTGCTGAGAATCTTGA | 296 | |
| *NFASC* | GAATCTGAGAGGGAGAGTTGC | GCGATCCTAAGCCTTCCCAG | 319 | |
| *SFMBT2* | AGAGAACTGAGGGGGGAGATGCCA | CCCAGATGACTCTTTCAGTCC | 451 | |

## Supplementary Table 1. PCR primer sequences

Amplicon size with a deletion (DEL) or unedited, wild-type (WT) is shown.
