# Supplementary material for: Whole genome sequencing of CCR5 CRISPR-Cas9-edited Mauritian cynomolgus macaque blastomeres reveals large-scale deletions and off-target edits
Source: Front Genome Ed. 2023 Jan 12;4:1031275. doi: 10.3389/fgeed.2022.1031275 (PMC9877282; doi:10.3389/fgeed.2022.1031275)
Supplement: Supplementary file 2 [file Table2.docx]

| **Chr** | **4-1** | **4-2** | **4-3** | **4-4** | **4-5** | **4-6** | **5-1** | **5-3** | **5-4** | **5-5** | **5-7** | **5-8** | **5-9** | **Dam** | **Sire** |
| --- | --- | --- | --- | --- | --- | --- | --- | --- | --- | --- | --- | --- | --- | --- | --- |
| 1 | 12.08 | 10.00 | 64.09 | 54.01 | 20.59 | 21.04 | 26.09 | 12.10 | 15.11 | 26.22 | 16.14 | 29.19 | 13.20 | 37.03 | 31.12 |
| 2* | 13.67 | 10.54 | 2.21 | 57.28 | 18.67 | 47.43 | 28.72 | 10.70 | 76.29 | 31.51 | 19.68 | 17.30 | 14.67 | 38.77 | 32.76 |
| 3 | 10.84 | 8.40 | 39.19 | 39.25 | 22.77 | 31.83 | 24.16 | 14.01 | 28.63 | 11.00 | 22.63 | 21.99 | 10.81 | 37.40 | 31.31 |
| 4 | 16.39 | 8.42 | 12.92 | 10.48 | 4.07 | 46.40 | 28.57 | 24.87 | 10.66 | 9.04 | 21.59 | 17.20 | 19.33 | 38.15 | 32.24 |
| 5 | 13.61 | 10.11 | 30.27 | 10.64 | 4.46 | 22.78 | 26.92 | 11.98 | 51.12 | 10.90 | 17.38 | 18.08 | 18.49 | 38.83 | 32.86 |
| 6 | 11.80 | 7.58 | 3.08 | 14.17 | 21.58 | 35.55 | 28.36 | 22.45 | 18.55 | 10.11 | 29.34 | 27.89 | 11.85 | 38.56 | 32.53 |
| 7 | 29.74 | 7.41 | 0.94 | 9.30 | 32.96 | 46.07 | 29.88 | 21.52 | 25.67 | 7.12 | 32.63 | 15.64 | 12.16 | 37.37 | 31.41 |
| 8 | 12.70 | 8.23 | 35.53 | 10.71 | 22.82 | 22.47 | 27.73 | 19.96 | 27.32 | 9.53 | 74.44 | 10.29 | 15.00 | 40.22 | 33.88 |
| 9 | 11.23 | 7.93 | 77.77 | 9.66 | 4.57 | 32.01 | 27.55 | 9.34 | 5.02 | 21.40 | 23.97 | 28.87 | 13.17 | 39.05 | 32.96 |
| 10 | 8.84 | 6.29 | 3.44 | 7.04 | 39.17 | 26.12 | 30.46 | 9.40 | 24.41 | 15.03 | 32.53 | 5.93 | 14.16 | 35.54 | 29.33 |
| 11 | 11.50 | 8.49 | 1.42 | 11.40 | 61.53 | 44.27 | 28.06 | 10.49 | 14.24 | 27.20 | 16.64 | 20.93 | 13.45 | 37.65 | 31.69 |
| 12 | 12.49 | 8.62 | 1.47 | 10.55 | 30.67 | 43.28 | 29.23 | 9.32 | 35.73 | 21.94 | 24.44 | 35.56 | 8.95 | 38.54 | 32.68 |
| 13 | 12.13 | 10.15 | 53.49 | 12.02 | 23.32 | 21.12 | 28.26 | 8.79 | 72.43 | 18.83 | 18.48 | 23.76 | 8.75 | 37.72 | 31.64 |
| 14 | 11.06 | 6.97 | 0.94 | 47.68 | 46.02 | 39.85 | 25.81 | 8.61 | 40.02 | 23.79 | 15.25 | 25.95 | 10.17 | 36.94 | 30.84 |
| 15 | 10.65 | 7.62 | 34.56 | 49.00 | 15.44 | 44.06 | 20.00 | 11.78 | 8.79 | 28.13 | 22.55 | 26.07 | 14.31 | 37.40 | 31.26 |
| 16 | 9.62 | 6.60 | 0.81 | 7.24 | 16.49 | 35.71 | 21.86 | 15.74 | 11.74 | 5.59 | 16.95 | 12.17 | 14.59 | 34.37 | 28.41 |
| 17 | 11.97 | 8.53 | 16.09 | 10.00 | 52.97 | 43.50 | 45.73 | 12.66 | 35.08 | 24.83 | 25.27 | 24.24 | 9.22 | 38.59 | 32.75 |
| 18 | 11.53 | 8.65 | 18.01 | 51.54 | 26.11 | 22.29 | 28.10 | 11.10 | 50.10 | 13.07 | 39.10 | 17.27 | 11.27 | 38.31 | 32.17 |
| 19 | 7.72 | 5.33 | 2.49 | 32.42 | 33.41 | 12.76 | 21.32 | 8.57 | 12.97 | 7.44 | 6.79 | 15.64 | 8.88 | 31.85 | 25.99 |
| 20 | 9.47 | 7.26 | 0.84 | 41.12 | 41.59 | 37.38 | 24.38 | 9.95 | 13.19 | 20.68 | 13.41 | 20.83 | 9.26 | 34.93 | 28.89 |
| X | 13.84 | 6.59 | 14.27 | 13.48 | 46.68 | 24.86 | 30.58 | 16.44 | 8.87 | 4.09 | 46.10 | 0.37 | 42.94 | 37.44 | 16.08 |
| MIT | 10749.3 | 78392.00 | 47944.70 | 65896.60 | 47796.70 | 17885.10 | 13173.00 | 7840.66 | 17679.90 | 15005.70 | 53353.00 | 18915.50 | 33320.30 | 278.26 | 188.71 |

## Supplementary Table 2. Sequencing coverage by sample and chromosome

Asterisk/shading denotes that *CCR5* is positioned on chr2. Abbreviations: Chr=chromosome, MIT= mitochondrial DNA.
