# Supplementary material for: Whole genome sequencing of CCR5 CRISPR-Cas9-edited Mauritian cynomolgus macaque blastomeres reveals large-scale deletions and off-target edits
Source: Front Genome Ed. 2023 Jan 12;4:1031275. doi: 10.3389/fgeed.2022.1031275 (PMC9877282; doi:10.3389/fgeed.2022.1031275)
Supplement: Supplementary file 3 [file Table5.docx]

|  | Blastomere | | On-target genotype | | On-target INDEL | On-target SV | Off-target INDEL  (validated by Sanger) | Off-target SV |
| --- | --- | --- | --- | --- | --- | --- | --- | --- |
|  |  |  | **PCR** | **WGS** |  |  |  |  |
| Embryo 4 | | 1 | HET DEL | |  |  |  | LIPC |
|  |  | 2 | WT | |  |  |  |  |
|  |  | 3 | no signal | low coverage, inconclusive |  |  | SFMBT2 | NFASC, SFMBT2, LIPC |
|  |  | 4 | HET DEL | |  | 2 INV  1 DEL |  | NFASC, LIPC |
|  |  | 5 | WT | |  | 1 INV | LIPC | LIPC |
|  |  | 6 | HOM DEL | |  | 1 DUP  1 DEL* | SFMBT2, LIPC | LIPC |
| Embryo 5 | | 1 | HOM DEL | |  |  | SFMBT2, LIPC | LIPC |
|  |  | 2 | no signal | low coverage, inconclusive |  |  |  |  |
|  |  | 3 | HOM DEL | low coverage, inconclusive |  | 1 INV | SFMBT2 | NFASC |
|  |  | 4 | HET DEL | HOM DEL | HOM insertions | 2 INV  1 DEL | SFMBT2 | NFASC |
|  |  | 5 | HOM DEL | |  | 1 INV  1 DEL | SFMBT2 | LIPC |
|  |  | 7 | no signal | low coverage, inconclusive |  |  | SFMBT2 | NFASC,LIPC |
|  |  | 8 | no signal | HOM DEL |  | 2 DEL | SFMBT2 | NFASC |
|  |  | 9 | no signal | low coverage, inconclusive |  |  |  | LIPC |

## Supplementary Table 5. Summary of on- and off-target outcomes in individual blastomeres.

Sample 5-2 WGS data was not included in the analysis due to an abnormal GC content. Asterisk denotes that the deletion was confirmed by PCR and gel electrophoresis.
