# Supplementary material for: Whole genome sequencing of CCR5 CRISPR-Cas9-edited Mauritian cynomolgus macaque blastomeres reveals large-scale deletions and off-target edits
Source: Front Genome Ed. 2023 Jan 12;4:1031275. doi: 10.3389/fgeed.2022.1031275 (PMC9877282; doi:10.3389/fgeed.2022.1031275)
Supplement: Supplementary file 4 [file Table3.docx]

| **Chr** | **4-1** | **4-2** | **4-3** | **4-4** | **4-5** | **4-6** | **5-1** | **5-3** | **5-4** | **5-5** | **5-7** | **5-8** | **5-9** |
| --- | --- | --- | --- | --- | --- | --- | --- | --- | --- | --- | --- | --- | --- |
| 1 | 851.8 | 565.7 | 88.1 | 758.5 | 685.1 | 959.1 | 310.4 | 231.5 | 204.3 | 176.2 | 131.6 | 241.4 | 99.0 |
| 2 | 657.1 | 527.9 | 136.2 | 661.6 | 611.0 | 510.8 | 321.8 | 260.7 | 380.4 | 278.5 | 134.3 | 78.8 | 122.8 |
| 3 | 730.7 | 514.8 | 140.2 | 693.7 | 623.2 | 708.9 | 242.9 | 139.2 | 234.6 | 317.8 | 144.7 | 211.0 | 108.5 |
| 4 | 854.8 | 581.8 | 372.9 | 778.7 | 498.0 | 642.7 | 395.9 | 268.0 | 366.4 | 428.7 | 156.4 | 254.9 | 103.8 |
| 5 | 1049.7 | 661.3 | 178.9 | 901.0 | 606.2 | 1098.5 | 411.2 | 216.1 | 341.2 | 203.0 | 179.2 | 342.9 | 123.8 |
| 6 | 877.3 | 601.8 | 191.6 | 804.8 | 337.7 | 821.7 | 338.0 | 351.1 | 267.2 | 78.4 | 148.3 | 236.2 | 120.5 |
| 7 | 760.8 | 523.0 | 144.7 | 697.2 | 203.4 | 727.3 | 422.7 | 356.4 | 232.7 | 69.4 | 164.9 | 283.2 | 125.4 |
| 8 | 786.3 | 612.2 | 162.3 | 752.1 | 312.1 | 897.8 | 290.8 | 324.0 | 62.0 | 78.7 | 227.8 | 76.9 | 151.1 |
| 9 | 798.5 | 568.3 | 295.5 | 716.9 | 462.9 | 1072.4 | 429.1 | 344.3 | 206.6 | 414.8 | 149.7 | 303.4 | 109.0 |
| 10 | 706.9 | 455.0 | 159.1 | 612.6 | 383.7 | 946.4 | 310.2 | 277.0 | 325.3 | 315.3 | 80.6 | 244.0 | 115.3 |
| 11 | 806.2 | 585.6 | 167.8 | 758.1 | 379.8 | 601.5 | 370.8 | 353.6 | 75.8 | 269.5 | 84.1 | 281.7 | 173.2 |
| 12 | 670.2 | 520.3 | 128.7 | 619.6 | 203.3 | 502.5 | 292.7 | 284.7 | 54.3 | 322.5 | 194.6 | 222.2 | 97.6 |
| 13 | 803.1 | 512.6 | 461.8 | 706.8 | 709.4 | 904.8 | 433.2 | 282.7 | 522.1 | 407.4 | 88.6 | 354.2 | 94.0 |
| 14 | 632.9 | 463.1 | 133.9 | 637.2 | 431.0 | 505.4 | 375.3 | 259.1 | 174.5 | 348.0 | 177.9 | 247.0 | 125.1 |
| 15 | 798.7 | 547.7 | 154.0 | 683.7 | 601.9 | 617.6 | 336.8 | 264.6 | 65.0 | 198.4 | 69.4 | 241.3 | 143.8 |
| 16 | 553.6 | 340.9 | 112.9 | 475.9 | 205.0 | 397.0 | 312.2 | 317.0 | 66.6 | 68.1 | 72.0 | 64.1 | 188.0 |
| 17 | 1238.9 | 796.8 | 592.0 | 1098.2 | 371.3 | 1000.9 | 333.7 | 403.8 | 509.6 | 464.5 | 192.3 | 291.7 | 124.2 |
| 18 | 851.5 | 582.7 | 198.0 | 724.7 | 583.4 | 950.2 | 368.6 | 305.6 | 169.4 | 177.2 | 123.5 | 344.2 | 105.5 |
| 19 | 583.2 | 439.0 | 155.7 | 710.3 | 365.5 | 697.4 | 394.8 | 276.8 | 406.3 | 229.0 | 99.4 | 246.2 | 163.5 |
| 20 | 895.9 | 677.4 | 143.0 | 800.3 | 398.4 | 696.3 | 400.9 | 331.4 | 54.0 | 328.5 | 59.3 | 313.8 | 130.9 |
| X | 609.7 | 430.7 | 241.7 | 611.1 | 401.9 | 662.2 | 92.3 | 110.7 | 95.7 | 181.3 | 127.4 | 132.3 | 88.4 |
| MIT | 3921.6 | 4042.3 | 12006.1 | 5309.3 | 5309.3 | 5429.9 | 8144.8 | 4343.9 | 4223.3 | 4283.6 | 3499.3 | 2956.3 | 6033.2 |

## Supplementary Table 3. The number of de novo single nucleotide variants in individual blastomeres that were not present in the parental sequence. The counts are normalized by the size of the chromosome (chr).
