# Supplementary material for: Whole genome sequencing of CCR5 CRISPR-Cas9-edited Mauritian cynomolgus macaque blastomeres reveals large-scale deletions and off-target edits
Source: Front Genome Ed. 2023 Jan 12;4:1031275. doi: 10.3389/fgeed.2022.1031275 (PMC9877282; doi:10.3389/fgeed.2022.1031275)
Supplement: Supplementary file 5 [file Table4.docx]

| Number of Mismatches | Number of Predicted Off-targets | |
| --- | --- | --- |
|  | gRNA 1 | gRNA 2 |
| 1 | 0 | 0 |
| 2 | 10 | 5 |
| 3 | 38 | 37 |
| 4 | 243 | 304 |
| 5 | 1,587 | 2,398 |
| 6 | 10,058 | 15,200 |
| 7 | 51,610 | 85,660 |
| 8 | 224,995 | 370,009 |
| 9 | 817,171 | 1,270,653 |

## Supplementary Table 4. The number of predicted off-target sites identified by Cas-OFFinder for each gRNA.
