# Supplementary material for: Whole genome sequencing of CCR5 CRISPR-Cas9-edited Mauritian cynomolgus macaque blastomeres reveals large-scale deletions and off-target edits
Source: Front Genome Ed. 2023 Jan 12;4:1031275. doi: 10.3389/fgeed.2022.1031275 (PMC9877282; doi:10.3389/fgeed.2022.1031275)
Supplement: Supplementary file 6 [file DataSheet1.docx]

**Supplementary File 2. Validation of sequence deletion by Sanger sequencing of PCR amplicons**

For all sequence alignments listed below, the top line is the expected amplicon sequence, and the bottom line is the sanger sequence.

5-4

Sample: 5-4 deletion

Chromosome: 2

Position: on-target between gRNAs

Deletion size: 198 bp

PCR amplicon size: with deletion 415 bp;

3 lower bands isolated from the gel as shown in adjacent figure

Alignment to sequence containing the expected deletion:

Band 1

69 GTGGGCAACATACTGGTCGTCCTCATCCTGATAAACTGCAAAAGGCTGAAAAGCATGACT 128

||||||| |||||||||||||||| |||||||||||||||||||||||||||||||||||

29 GTGGGCA-CATACTGGTCGTCCTCNTCCTGATAAACTGCAAAAGGCTGAAAAGCATGACT 87

129 GACATCTACCTGCTCAACCTGGCCATCTCTGACCTGCTTTTCCTTCTTACTGTCCCCTTC 188

||||||||||||||||||||||||||||||||||||||||||||||||||||||||||||

88 GACATCTACCTGCTCAACCTGGCCATCTCTGACCTGCTTTTCCTTCTTACTGTCCCCTTC 147

189 TGGGCTCACTATGCTGCTGCCCAGTGGGAC 218

||||||||||||||||||||||||||||||

148 TGGGCTCACTATGCTGCTGCCCAGTGGGAC 177

-deleted region (198 bp)-

416 CCCAGGAATCATCTTTACCAGATCTCAGAGAGAAGGTCTTCATTACACCTGCAGCTCTCA 475

||||||||||||||||||||||||||||||||||||||||||||||||||||||||||||

177 CCCAGGAATCATCTTTACCAGATCTCAGAGAGAAGGTCTTCATTACACCTGCAGCTCTCA 236

476 TTTTCCATACAGTCAGTATCAATTCTGGAAGAATTTTCAGACATTAAAGATGGTCATCTT 535

||||||||||||||||||||||||||||||||||||||||||||||||||||||||||||

237 TTTTCCATACAGTCAGTATCAATTCTGGAAGAATTTTCAGACATTAAAGATGGTCATCTT 296

536 GGGGCTGGTCCTGCCGCTGCTTGTCATGGTCATCTGCTACTCGGGAATCCTGAAAACTCT 595

||||||||||||||||||||||||||||||||||||||||||||||||||||||||||||

297 GGGGCTGGTCCTGCCGCTGCTTGTCATGGTCATCTGCTACTCGGGAATCCTGAAAACTCT 356

596 GCTTCGGTGTCGAAACGA 613

||||||||||||||||||

357 GCTTCGGTGTCGAAACGA 374

Band 2

55 TCATCTTTGG-TTTTGTGGGCAACATACTGG-TCGTCCTCATCCTGATAAACTGCAAAAG 112

|||||||||| |||||||||||||||||||| ||||||||||||||| ||||||||||

20 TCATCTTTGGNTTTTGTGGGCAACATACTGGATCGTCCTCATCCTGANCNACTGCAAAAG 79

113 GCTGAAAAGCATGACTGACATCTACCTGCTCAACCTGGCCATCTCTGACCTGCTTTTCCT 172

||||||||||||||||||||||||||||||||||||||||||||||||||||||||||||

80 GCTGAAAAGCATGACTGACATCTACCTGCTCAACCTGGCCATCTCTGACCTGCTTTTCCT 139

173 TCTTACTGTCCCC-TTC-TGGGCTCACTATGCTGCTGCCCAGTGGGAC 218

||||||||||||| ||| ||||||||||||||||||| || || |||

140 TCTTACTGTCCCCGTTCGTGGGCTCACTATGCTGCTGGCCTNTGNGAC 187

-deleted region (201 bp)-

419 AGGAATCATCTTTACCAGATCTCAGAGAGAAGGTCTTCATTACACCTGCAGCTCTCATTT 478

|||||| | || || ||||| | | | | |||| | || || | | || |

190 AGGAATNANCTNTANNTGATCTNTTAANGNANGNNGTCATNANACGTGNANNNCNNATNT 249

479 TCCATACAGTCAGTATCAATTCTGGAAGAATTTTCAGACATTAAAGATGGTCATCTTGGG 538

| || | | || | || || |||| | || | | || || |||| ||||||| |

250 TNCANAGAANNAGAAGCACNTCNGGAAAANTTNNCNAANATGNAAAATGGACATCTTGAG 309

539 GCTGGTCCTGCCGCTGCTTGTCATGGTCATCTGCTACTCG-GGAATCCT-GAAAACTCTG 596

||||| || |||| || | || | |||| ||| | ||||||| ||||||| |

310 GCTGGNNNNGCTGCTGGNTGNCNTGANCNGCTGCAACTNGAAGAATCCTGGAAAACTNNG 369

597 C-TTCGGTGTCGAAA 610

| ||||||| | |||

370 CGTTCGGTGACCAAA 384

Band 3

49 TGGTGTTCATCTTTGGTTTTGTGGGCAACATACTGGTCGTCCTCATCCTGATAAACTGCA 108

||||||||||||||||||||||||||||||||||||||||||||||||||| |||||||

15 TGGTGTTCATCTTTGGTTTTGTGGGCAACATACTGGTCGTCCTCATCCTGANNAACTGCA 74

109 AAAGGCTGAAAAGCATGACTGACATCTACCTGCTCAACCTGGCCATCTCTGACCTGCTTT 168

||||||||||||||||||||||||||||||||||||||||||||||||||||||||||||

75 AAAGGCTGAAAAGCATGACTGACATCTACCTGCTCAACCTGGCCATCTCTGACCTGCTTT 134

169 TCCTTCTTACTGTCCCCTTCTGGGCTCACTATGCTGCT 206

||||||||||||||||||||||||||||||||||||||

135 TCCTTCTTACTGTCCCCTTCTGGGCTCACTATGCTGCT 172

-deleted region (208 bp)-

414 CTCCCAGGAATCATCTTTACCAGATCTCAGAGAGAAGGTCTTCATTACACCTGCAGCTCT 473

|||| ||| || ||||||||| |||||||||| ||||||||||||||||||||||||||

549 CTCCAAGGTATGCTCTTTACCANATCTCAGAGANAAGGTCTTCATTACACCTGCAGCTCT 608

474 CATTTTCCATACAGTCAGTATCAATTCTGGAAGAATTTTCAGACATTAAAGATGGTCATC 533

|||||||||||||||||||||| |||||||||||||||||||||||||||||||| ||||

609 CATTTTCCATACAGTCAGTATCNATTCTGGAAGAATTTTCAGACATTAAAGATGGNCATC 668

534 TTGGGGCTGGTCCTGCCGCTGCTTGTCATGGTCATCTGCTACTCGGGAATCCTGAAAACT 593

||||||||||||||||||||||||||||||| ||||||||||||||||||||||||||||

669 TTGGGGCTGGTCCTGCCGCTGCTTGTCATGGGCATCTGCTACTCGGGAATCCTGAAAACT 728

594 CTGCTTCGGTGTCGAAACGA 613

||||||||||||| ||||||

729 CTGCTTCGGTGTCNAAACGA 748

Sample: 4-6 deletion

Chromosome: 2

Position: 101,400,160-101,400,916

Deletion size: 756 bp

PCR amplicon size: with deletion 2,156

Alignment to sequence containing the expected deletion:

1129 AAAATGTGAAAAATATTT---CCTGCCTTATAA--GGTTGCCCT--GAGG-ATTAAATG- 1179

|||||| |||||| |||| | ||||| || | || ||| |||| ||||||||

1063 AAAATGNGAAAAAAATTTTTCNTTCCCTTAAAAAGGNTTNCCCCTGGAGGGATTAAATGN 1004

1180 AAT-GAATGGG-TATGAC-TCTTAGA-CAGTGA-TTGGCATCC--AGTATGTGCCCTC-G 1231

||| ||||||| |||| | ||||||| |||||| ||| ||| | ||||||||||||| |

1003 AATNGAATGGGNTATGGCNTCTTAGAACAGTGANTTGNCATTCCAAGTATGTGCCCTCNG 944

1232 AGGT-CTCTTAATTATTAC-TGACTT-GCTC--ATAGTGCATGCTCTTTGTGGACTAACT 1286

|| | || |||||| || ||| || ||| |||| ||||||| ||||||| ||||||

943 AGNTTCTTTTAATTTTTTNATGAATTTGCTTCAATAGNGCATGCTNTTTGTGGNCTAACT 884

1287 -CCAGAGAATGGCAGCATC--AATAAACATCTT-AAGACTGAGTTGCAG--CCGGGCA-T 1339

|||||||||||||||||| | ||| |||||| || ||||||||||| ||||||| |

883 TCCAGAGAATGGCAGCATCCAANTAACCATCTTTAANACTGAGTTGCAAGCCCGGGCAAT 824

1340 GGTGGCTCATGTTTGCAATCCC-AGCATTCT-AGGA-GGCTGAGG-CAGGAGGATTGCTT 1395

|||||||||||||||||||||| |||||||| |||| || | | | ||||||||||||||

823 GGTGGCTCATGTTTGCAATCCCCAGCATTCTNAGGAAGGNTAANGNCAGGAGGATTGCTT 764

1396 GAGCCCAGGAGTTCGAGACCAGCCTGGGCAACATAGTGTGATCTTGTCTCTATAAAAATA 1455

|||||||||||||| |||||||||||||||||||||||||||||||||||||||||||||

763 GAGCCCAGGAGTTCAAGACCAGCCTGGGCAACATAGTGTGATCTTGTCTCTATAAAAATA 704

1456 AACAAAATTAGGCCAGGCGTGGTGGCTTACGCCTGTAATCCCAGCACTTTGGGAGGCCAA 1515

||||||||||||||||||||||||||||||||||||||||||||||||||||||||||||

703 AACAAAATTAGGCCAGGCGTGGTGGCTTACGCCTGTAATCCCAGCACTTTGGGAGGCCAA 644

1516 GGCAGGCAGATCACAGGGTCAGGAGATGGAGACCATCCTGGCTAACACGGTGAAACCCCA 1575

||||||||||||||||||||||||||||||||||||||||||||||||||||||||||||

643 GGCAGGCAGATCACAGGGTCAGGAGATGGAGACCATCCTGGCTAACACGGTGAAACCCCA 584

1576 TCTCTACTAAAAATACAAAAACAAAATTAGCCGGGTGTGGTGATGGGTGCCTGTAGTCCC 1635

||||||||||||||||||||||||||||||||||||||||||||||||||||||||||||

583 TCTCTACTAAAAATACAAAAACAAAATTAGCCGGGTGTGGTGATGGGTGCCTGTAGTCCC 524

1636 AGCTACTCAGGAGGCTGAGGCAGAAGAATGGCGTGAACCCAGGAGGAGGAGCTTGCAGTG 1695

|||||||||||||||||||||||||||||||| |||||||||||||||||||||||||||

523 AGCTACTCAGGAGGCTGAGGCAGAAGAATGGCATGAACCCAGGAGGAGGAGCTTGCAGTG 464

1696 AGTTGAGATCGCGCCACTGCACTCCAGCCTGGGCAAAAGAGCAAGACTCCATCTC----- 1750

|||||||||||||||||||||||||||||||||||||||||||||||||| ||||

463 AGTTGAGATCGCGCCACTGCACTCCAGCCTGGGCAAAAGAGCAAGACTCCGTCTCAAATA 404

1751 ---------------aaataaataaatacataaataacaataaaaataaacaaaatTAGC 1795

||||||||||||| |||||||||||||||||||||||||||||||

403 AATAAATAAATAAATAAATAAATAAATAAATAAATAACAATAAAAATAAACAAAATTAGC 344

1796 TGTTACAGGTGTGGTGGCACCTGTAGTCCCCAGCCACTTGGAAAGGTGAGGTGAGAGGAT 1855

||||||||||||||||||||||||||||||||||||||||||||||||||||||||||||

343 TGTTACAGGTGTGGTGGCACCTGTAGTCCCCAGCCACTTGGAAAGGTGAGGTGAGAGGAT 284

1856 TGCTTGAGCCCGGGAGGTCCAGGCTTCAGTGAACCATGATCGAGCCACTGCACTCCAGCC 1915

|||||||||||||||||||||||||||||||| |||||||||||||||||||||||||||

283 TGCTTGAGCCCGGGAGGTCCAGGCTTCAGTGATCCATGATCGAGCCACTGCACTCCAGCC 224

1916 TGAGCGACAGAGTGAGACCCTGTCTCAAAACAACGACAACAGCAACAACAAAAAGGCTGA 1975

||||||||||||||||||||||||||||||||||||||||||||||||||||||||||||

223 TGAGCGACAGAGTGAGACCCTGTCTCAAAACAACGACAACAGCAACAACAAAAAGGCTGA 164

1976 GCTGCA-CCATGCTTGACCCAGTTTCTTAAAATTGTTATCAAAGCTTCATTCACTCTGTG 2034

|||||| ||||||||||||||||||||||||||||||||||||||||||||| |||||||

163 GCTGCATCCATGCTTGACCCAGTTTCTTAAAATTGTTATCAAAGCTTCATTCTCTCTGTG 104

2035 GTGCTATAGAGCACAAGATTTT 2056

||||||||||||||||||||||

103 GTGCTATAGAGCACAAGATTTT 82

Sample: 5-5 deletion

Chromosome: 2

Position: 101,395,673-101,400,914

Deletion size: 5.24 kb

PCR amplicon size: with deletion 499 bp

Alignment to sequence containing the expected deletion:

61 CCCTCCAATATGGGCATGGAGTCTAGAGTGACAAACTGATCAAAAGTTCATTTCCCATGG 120

||||||||||||||||||||||||||||||||||||||||||||||||||||||||||||

14 CCCTCCAATATGGGCATGGAGTCTAGAGTGACAAACTGATCAAAAGTTCATTTCCCATGG 73

121 GGAGTCCGAACATATTTAATAATAAAAAGAGAACAAGAGCCATGCAAACTGAGAAGGACA 180

||||||||||||||||||||||||||||||||||||||||||||||||||||||||||||

74 GGAGTCCGAACATATTTAATAATAAAAAGAGAACAAGAGCCATGCAAACTGAGAAGGACA 133

181 AAGTAGAAAGAGTAGCAGACACCAAGCAACTAAATCACAGCGTGATAAGCTGCTGGCTTG 240

||||||||||||||||||||||||||||||||||||||||||||||||||||||||||||

134 AAGTAGAAAGAGTAGCAGACACCAAGCAACTAAATCACAGCGTGATAAGCTGCTGGCTTG 193

241 TTGCCATTATTGTATCCAGAACATTTTATTTAAATGCTGAAGAATTTCCCATGGGTCCCC 300

||||||||||||||||||||||||||||||||||||||||||||||||||||||||||||

194 TTGCCATTATTGTATCCAGAACATTTTATTTAAATGCTGAAGAATTTCCCATGGGTCCCC 253

301 ACTTTCTTGTGAA----CTTTACCAGATCTCAGAGAGAAGGTCTTCATTACACCTGCAGC 356

||||||||||||| |||||||||||||||||||||||||||||||||||||||||||

254 ACTTTCTTGTGAATCATCTTTACCAGATCTCAGAGAGAAGGTCTTCATTACACCTGCAGC 313

357 TCTCATTTTCCATACAGTCAGTATCAATTCTGGAAGAATTTTCAGACATTAAAGATGGTC 416

||||||||||||||||||||||||||||||||||||||||||||||||||||||||||||

314 TCTCATTTTCCATACAGTCAGTATCAATTCTGGAAGAATTTTCAGACATTAAAGATGGTC 373

417 ATCTTGGGGCTGGTCCTGCCGCTGCTTGTCATGGTCATCTGCTACTCGGGAATCCTGAAA 476

||||||||||||||||||||||||||||||||||||||||||||||||||||||||||||

374 ATCTTGGGGCTGGTCCTGCCGCTGCTTGTCATGGTCATCTGCTACTCGGGAATCCTGAAA 433

477 ACTCTGCTTCGG 488

||||||||||||

434 ACTCTGCTTCGG 445

Sample: 5-8 deletion

Chromosome: 2

Position: 101,395,673-101,400,914

Deletion size: 5.24 kb

PCR amplicon size: with deletion 499 bp

Alignment:

Sanger sequence reaction did not perform well, and no alignments could be performed.

Sample: 5-8 deletion

Chromosome: 2

Position: 101,400,695-101,401,620

Deletion size: 925 bp

PCR amplicon size: without deletion 1,409; with deletion 484 bp

Alignment to sequence containing the expected deletion:

108 AAAAGGCTGAAAAGCATGACTGACATCTACCTGCTCAACC-TGGCCA-TCTCTGACCTGC 165

||| || | ||| || |||||| | || | |||||| ||| || ||||||| ||||

80 AAAGGGTTTAAANGCGGGACTGAGANCTTNAAGNTCAACCNTGGACAGTCTCTGAGCTGC 139

166 TTTTCCTTCTTAC-TGTCCCCTTCTGG--GCTCACTATGCTGCT 206

|||||| ||||| ||||||| || |||||||||||||||

140 TTTTCCNNCTTACATGTCCCCGGGCGGAGGCTCACTATGCTGCT 183

-deleted region-

1132 TTTATTTGGCATCTGTTTAAAGCAGATTTGATCTTTTAAGCCCATCAATTATAGAAAGCC 1191

|||| ||| ||||| |||||||||||| | ||||| |||||||| | ||| ||||||

186 TTTAGTTGNNATCTGNTTAAAGCAGATTNGNNCTTTTGAGCCCATCCANTATNNAAAGCC 245

1192 AAATCAAAATATGTTGATGAAAAATAGCAACCTTTTTATCTCTCCTTCACATGCATCAAG 1251

|||||| | | ||||||||||| | | ||||| ||||||||||||||||||||||

246 NAATCAANANNTNTTGATGAAAAAGATCNNCCTTTNNATCTCTCCTTCACATGCATCAAN 305

1252 TTATTGACAAACTCTCCCTTCTCTCCAAAAGTTCCTTATATATATTAAAAAGAAAGCCTC 1311

||||||||||| |||||||||||||||||||||| |||||||| |||| |||| || ||

306 TTATTGACAAANTCTCCCTTCTCTCCAAAAGTTCNNTATATATANTAAANAGAANGCNTC 365

1312 AGAGAATTGCTGATTCTTGAGTTTAGTGACCTGGACAGAAATACCAAAATTATTTCAGAA 1371

| | ||||||||||||||||||||||||||||||||||||||||| ||| |||||| |||

366 ATATAATTGCTGATTCTTGAGTTTAGTGACCTGGACAGAAATACCNAAANTATTTCNGAA 425

1372 ATGTACAACTTTTTACCTTGTACAAGGCAACATATGGG 1409

| |||||||||| |||||||||||||||||||||||||

426 ANGTACAACTTTGTACCTTGTACAAGGCAACATATGGG 463
