# Supplementary figures and images for: Whole genome sequencing of CCR5 CRISPR-Cas9-edited Mauritian cynomolgus macaque blastomeres reveals large-scale deletions and off-target edits
Source: Front Genome Ed. 2023 Jan 12;4:1031275. doi: 10.3389/fgeed.2022.1031275 (PMC9877282; doi:10.3389/fgeed.2022.1031275)

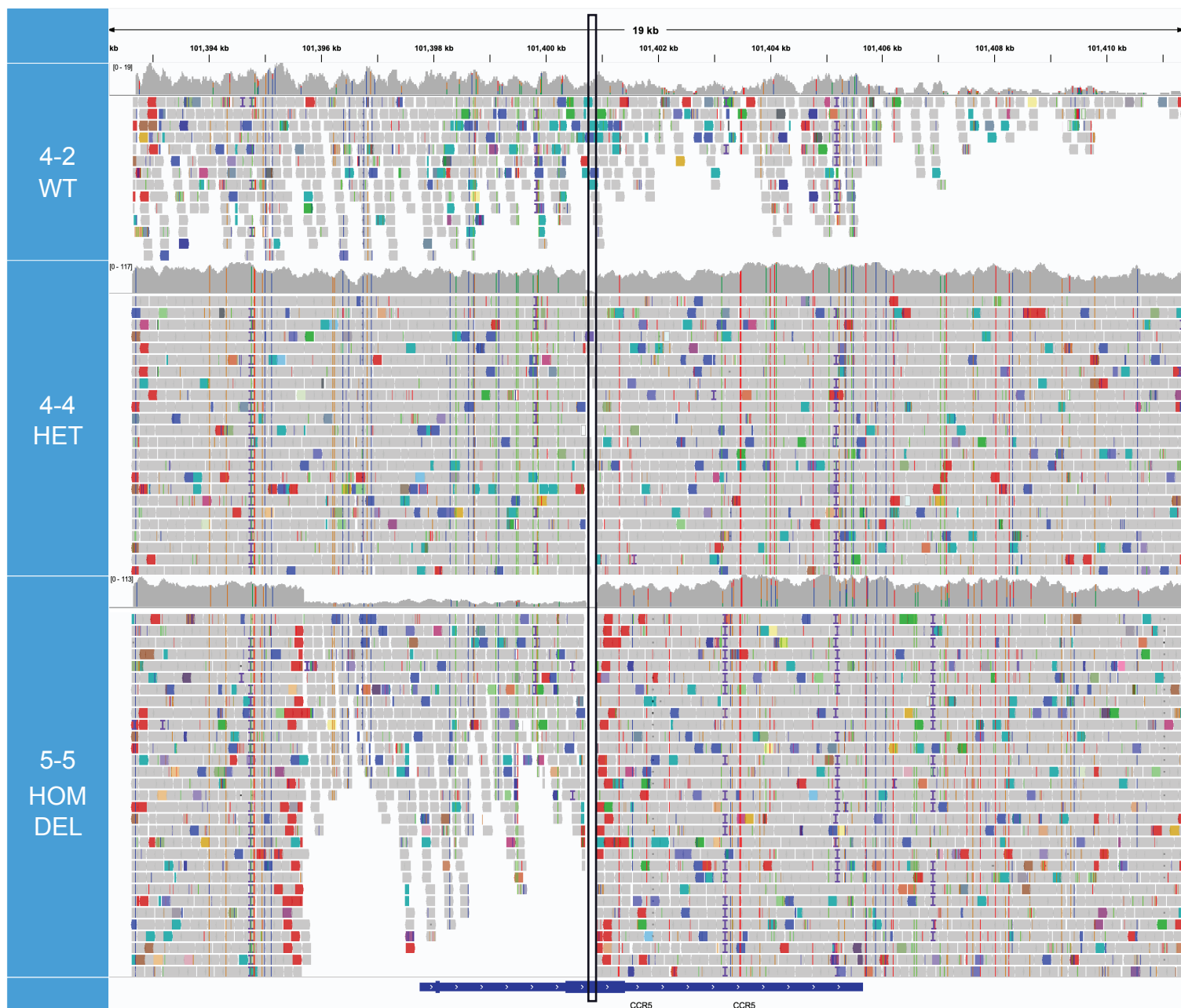

Supplement: Supplementary file 7 [file Image1.pdf]
